# Buffering Effects of Racial Discrimination on School Engagement: The Role of Culturally Responsive Teachers and Caring School Police

**DOI:** 10.1111/josh.12967

**Published:** 2020-11-12

**Authors:** Jessika H. Bottiani, Heather L. McDaniel, Lora Henderson, Jasmin E. Castillo, Catherine P. Bradshaw

**Affiliations:** ^1^ Assistant Professor, University of Virginia, School of Education and Human Development, PO Box 400281, Charlottesville, VA 22904.; ^2^ Assistant Professor, (hm8tc@virginia.edu), University of Virginia, School of Education and Human Development, PO Box 400281, Charlottesville, VA 22904.; ^3^ Youth‐Nex Postdoctoral Research Associate, (ljh5sk@virginia.edu), University of Virginia, School of Education and Human Development, PO Box 400281, Charlottesville, VA 22904.; ^4^ Doctoral Student, (jec542@psu.edu), Pennsylvania State University, Human Development and Family Studies, 27 Health and Human Development Building, University Park, PA 16802.; ^5^ Professor and Senior Associate Dean, (catherine.bradshaw@virginia.edu), University of Virginia, School of Education and Human Development, PO Box 400270, Charlottesville, VA 22904.

**Keywords:** school engagement, cultural responsiveness, school resource officers, school police, school aggression, family racial socialization

## Abstract

**BACKGROUND:**

Urban black adolescents' wellbeing in the early high school years can be negatively impacted by exposure to racial discrimination. These impacts may be buffered by supportive relationships with adults at school. We considered both the protective and promotive effects of culturally responsive teachers and caring school police on school engagement for students exposed to racial discrimination across settings.

**METHOD:**

This study leveraged baseline student report from a sample of urban, predominantly black high school students with elevated teacher‐rated levels of aggressive behavior (N = 397 9th graders; 91.2% black; 50.4% male; *J* = 10 schools). Using a path model with full‐information maximum likelihood estimation, we examined the associations of racial discrimination, teacher cultural responsiveness, and school police caring in relation to school engagement and school disconnection, adjusting for covariates, including family racial socialization.

**RESULTS:**

Frequency of racial discrimination was significantly associated with lower school engagement and greater school disconnection. Teacher cultural responsiveness was significantly, favorably associated with all outcomes. Police caring had no significant direct associations; however, there were moderation effects. When police caring was below average, increased racial discrimination was associated with significantly poorer attitudes toward school.

**CONCLUSIONS:**

Findings suggested that students' perceptions of school police caring may buffer links between racial discrimination experiences and school disconnection. Moreover, students who perceive that their teachers are culturally responsive may feel more engaged at school. Interventions to promote teachers' and school police officers' cultural responsiveness and caring may improve engagement among at‐risk urban youth who experience racial discrimination.

High rates of exposure to racial discrimination can adversely affect mental health^1^, ^2^ and hinder students' motivation to engage at school.^3^ Moreover, students with elevated risk of behavior problems are particularly likely to perceive their school as less supportive^4^; for urban youth of color, institutional, and interpersonal barriers, such as discrimination experiences, can impede equitable access to developmentally healthy and safe contexts for learning. The transition from middle to high school is a particularly challenging time for urban black and Latinx adolescents, impacting academic and social‐emotional functioning.^5^ As such, urban youth of color transitioning to high school and identified with elevated rates of behavior problems are a group likely to be vulnerable to negative schooling experiences.

However, relationships with supportive adults at school may buffer the effects of exposure to racial discrimination through warmth and sensitivity to students' experiences, diverse identities, and lives outside of school in their homes and communities. Specifically, supportive adults at school may leverage strengths and funds of knowledge local to their students' family and neighborhood life to support student engagement.^6^ Engagement has been linked to a range of student academic, social, and behavioral outcomes.^7^ In recognition of the importance of schools and school staff in promoting health and educational equity, child‐centered frameworks draw attention to the need for evidence‐based approaches to support and sustain wellbeing particularly among those at greater risk of adverse schooling experiences. The Whole School, Whole Community, Whole Child (WSCC) model, developed through a partnership between the US Centers for Disease Control and Prevention (CDC) and the ASCD (formerly the Association for Supervision and Curriculum Development),^8^ is one such framework that promotes a collaborative approach to improve child wellbeing.

The current study applied the WSCC framework in exploring equity‐related school supports by teachers and school police in a sample of predominantly urban black ninth‐graders with elevated rates of behavior problems. Given the prevalence of exposure to racial discrimination in this population,^9^ we were particularly interested in whether teacher cultural responsiveness and school police caring were associated with school engagement (promotive) and buffered the adverse impacts of exposure to racial discrimination (protective). We considered the potential buffering effects of non‐parental, school staff relationships (ie, teachers and caring school police), over and above home‐based supports, like positive family racial socialization among urban students of color with elevated aggressive or disruptive behaviors.^3^, ^10^ Our research hypotheses and corresponding model building were driven by theories of positive youth development, developmental resilience, and developmental systems theories.^11^, ^12^, ^13^ Consistent with these perspectives, we took a strength‐based approach to understand how youth might achieve a positive developmental outcome, school engagement, even when experiencing adversity (ie, racial discrimination). Based in developmental systems theories, we also explored social‐ecological contextual factors in the school and family settings that could serve as protective or promotive factors. We anticipated that these protective factors would be especially important for students who experienced discrimination,^14^, ^15^ but they may also have a promotive effect for students in these settings, regardless of adverse experiences like discrimination.

## Youth Exposure to Discrimination

Research suggests that 70% of urban, youth of color report experiencing some form of discrimination and over 40% of those experiences were perceived as at least “somewhat disturbing.”^16^ Racial discrimination across settings has been linked with negative outcomes at school including poorer academic motivation, engagement,^17^, ^18^ and school bonding.^19^ Perceived discrimination specifically at school has been linked with poorer academic performance, including lower grade point averages (GPAs) and student engagement,^20^ as well as an increased likelihood of high school dropout.^21^ This research highlights a clear relationship between discrimination and important school outcomes, regardless of whether discrimination was experienced within or outside of the school setting. This points to a corresponding need for educators to mitigate the impacts of racial discrimination to educate and care for the whole child and ultimately achieve their educational mission. Although some studies have examined students' personal experiences of societal discrimination as it relates to their schooling,^3^ this research is relatively limited, particularly among students with elevated risks of aggression. Given that discrimination has been associated with increased externalizing symptoms,^22^ it is critical to not only prevent racial discrimination experiences before they occur, but identify potential buffers that can offset the harmful impacts of these damaging experiences.

## Supportive Relationships with Adults

Healthy relationships with adults in the lives of children and youth provide an essential protective and promotive developmental function^23^; they appear to be particularly important for adolescents of color in the context of adverse life experiences like discrimination.^24^ For example, family racial socialization messages (ie, caregivers' messages of racial pride and preparation for bias), have been found to be critical in mitigating the negative impacts on self‐esteem of youth's experiences of racism.^25^ However, research also suggests the significant role of relationships with non‐parental adults as protective.^26^ Given the amount of time youth spend at school, it is likely that adults at school can play an important role in buffering some of the effects of discrimination on important youth outcomes, such as school engagement. In particular, 2 groups of adults at school—teachers and school police—may have the potential to buffer some of these experiences for urban youth of color.

## Culturally Responsive Teachers

Positive teacher‐student relationships in adolescence have been linked with student engagement and demonstrative protective effects on depressive symptoms and behavioral misconduct.^27^, ^28^ Teachers who engage in culturally responsive practices (CRPs) are theorized to promote student engagement,^29^ in part by engendering caring, reciprocal relationships with their students.^30^ CRPs include approaches that tap students' cultural and contextual funds of knowledge as rich resources for learning^31^ and teaches “to and through [students']…cultural strengths,”^6^ (p. 32). Empirical research regarding teachers' use of CRPs is somewhat limited, but available research supports its positive associations with observed student behavior, particularly when combined with proactive behavior supports.^32^ For example, students in classrooms led by culturally competent teachers were found to have more inclusive mutual friendships than their peers in classrooms with teachers who do not utilize CRPs.^33^ Although evidence on the effects of interventions to promote teacher CRP is also scant,^34^ some new studies are emerging.^35^ One such study showed that teachers who received professional development and coaching in CRPs demonstrated improvement in independently observed classroom interactions with students.^36^ Yet, much of the research on CRPs has focused on links to its promotive potential; few studies have examined whether CRPs can mitigate the effects of adverse experiences like racial discrimination on school engagement.

## Caring School Police

School police officers could also play an important role in buffering or exacerbating the negative impacts of discrimination. The number of schools with law enforcement officers on‐site has been increasing since the 1970s, when only 1% of US schools had a stationed police officer.^37^ By 2014 (most recently available data from the US Civil Rights Data Collection), the majority of US high schools had school police officers on‐site (67%).^38^ Amidst increasing police presence in the schools, the National Association of School Resource Officers^39^ developed a model for school police that focuses on school police roles in the school as informal counselors, collaborative educators (educating adults at the school on school safety), and law enforcement officers protecting children. Yet, findings on the effects of school police presence on student safety, engagement, and other outcomes have been mixed; some studies suggest that their presence has no effect on students' school behavior,^40^ whereas other studies show that students' interactions with school police increase positive attitudes about the school police officers, but actually decrease their feelings of school connectedness.^41^ Observations of school police presence have also been linked to increased students' perceptions of safety, though with some indication this was less so the case for black students.^42^ A recent longitudinal study found that increased school police presence over time was not associated with improved school safety, but was linked to increases in exclusionary school discipline.^69^ Given the mixed findings, continued presence of officers at schools, and growing controversy regarding the role of school police, particularly in relation to black youth, additional research is needed to understand whether and how school police officers may be protective or promotive of school engagement.

## The Current Study

The current study sought to explore how experiences of discrimination are associated with school engagement and disconnection for urban predominantly black youth identified as demonstrating elevated disruptive behavior. The data come from a larger study in which students were screened in the fall of their ninth‐grade year by their teachers (eg, language arts) to identify those students with elevated levels of aggressive behavior. Eligible students recruited into the project then completed a written self‐report measure of their attitudes and experiences related to school. Given the heightened concerns about the outcomes for this population of high‐risk youth, including potential increased negative police contact^43^ and that perceived discrimination from non‐school sources are still relevant to students' schooling experiences,^20^ we explored how supportive school practices, namely teacher cultural responsiveness and school police caring, might buffer the negative impacts of discrimination on school engagement. Specifically, we were interested in whether higher levels of discrimination (including personal and societal experiences) were associated with lower school engagement and elevated school disconnection. In addition, we were interested in the extent to which of these associations were moderated by perceived teacher cultural responsiveness or school police officer caring, grounded in theory on positive youth development, resilience, and developmental systems theory.^11^, ^12^, ^13^ Students engaging in problem behaviors at school may have disproportionate interactions with school police officers, which presents school police officers with the opportunity to build a positive, protective relationship with these youth to shield them from other negative influences, such as the impacts of discrimination. Given the complexity of these associations, we considered these relations while controlling for family racial socialization and youth demographic characteristics, which also likely to play a role in this dynamic. We hypothesized that perceived discrimination would be associated with lower levels of school engagement and increased disconnection, and that teachers' use of CRPs and relationships with caring school police officers would attenuate the association between perceived discrimination and both engagement and disconnection.

## METHODS

### Participants

Survey data were collected at baseline from 397 students participating in the larger study. Of these, 91.2% of participants were African‐American or black, 1.3% where white, and 1.8% were Latinx or Hispanic. Additional student demographics and school characteristics are provided in Table 1.

**Table 1:** Demographics of Participating Students and Schools (N = 397, J = 10)

Student Demographics	N	Percentage
Gender		
Female	197	49.6
Male	200	50.4
Race		
Black	362	91.2
American Indian or Native American	4	1
White	5	1.3
Latinx or Hispanic	7	1.8
White	16	.8
Not reported	3	.8

School Demographics	M (SD)	Range
Total enrollment	943.1 (446.2)	404–1663
Black students (%)	88.9 (10.3)	69.6–98.1
Free/reduced priced meals (%)	56.2 (12.0)	29.0–72.9
Attendance (%)	79.5 (9.14)	61.2–93.7
Out‐of‐school suspensions (%)	11.7 (8.7)	.71–28.0

### Measures

#### *Discrimination*

Student frequency of *racial discrimination* experiences were measured using 5 selected items from the Racism and Life Experiences Scales.^44^ Students were asked to think about experiences involving racism, racial discrimination, or racial prejudice during the 6 months prior to completing the survey and then they were asked questions about the frequency of those events. Items included assessment of the frequency of personally experienced racial discrimination; personally witnessing racial discrimination against someone else; hearing about someone else's experience of discrimination; observing limited participation in decision‐making, opportunities, and access to resources for those in their racial group; and, general frequency of hearing about incidents of racial discrimination from family, friends, and neighbors. These items were answered on a 5‐point Likert‐scale (never to very often; 5‐item α = .86).

#### *School engagement*

Two scales from the Maryland Safe and Supportive Schools Survey (MDS3^45^) measured school engagement in this study. The MDS3 Survey is a comprehensive assessment of school climate that includes subscales measuring engagement. Four items measured *academic engagement* ^46^ as perception of academic success and importance of finishing high school (4‐item α = .81). Schools' *culture of equity* ^46^ was measured by perceived fairness at the school regardless of race, gender, and socio‐economic status, and whether the school provided culturally, racially, and ethnically representative educational materials (4‐item α = .79). All answer choices were on a 4‐point Likert‐scale (*strongly disagree* to *strongly agree*), whereby a high score represented more favorable school engagement. Prior research has demonstrated that this scale has strong psychometric properties and is robust with regard to measurement invariance.^47^, ^48^

#### *School disconnection*

The Behavior Assessment System for Children, Second Edition (BASC‐2; Adolescent [ages 12‐21])^49^ is a widely used self‐report measure to assess behavioral and emotional functioning. The reliability and validity of the BASC‐2 have been studied extensively. Two clinical subscales from the BASC‐2 Adolescent Self‐Report measure, Attitude to School (9‐item α = .78) and Attitude to Teachers (7‐item α = .73) were used to assess aspects of *school disconnection*. These items assessed positive and negative experiences at school or with teachers and were answered on a 4‐point Likert‐type scale (never to almost always), with positively worded items reverse coded. As such, higher scores on these scales were associated with poorer attitudes toward school and teachers. On the clinical scales of the BASC‐2, scores in the 60‐70 range are considered “at‐risk” and scores above 70 are “clinically significant”.

#### *Caring and culturally responsive relationships*

Student perceptions of *teacher CRPs* were measured using 4 items adapted from the cultural socialization subscale of a school racial climate measure^50^ and 4 items from the Maryland's Safe and Supportive Schools Initiative Student Survey (MDS3).^45^ Participants answered questions such as, “my teachers ask me about my culture and what it means to me” and “my teachers encourage me to do assignments or reports on people from diverse race and ethnicities,” on a 4‐point Likert‐scale (*strongly agree* to *strongly disagree*), whereby items were scored such that higher scores were associated with more student perceived CRPs (4‐item α = .79). Student perceptions of *school police officer caring* were adapted from the teacher connectedness scale of the MSD3 survey.^45^ Students were asked to respond to statements such as “Our school's police officer treats me respectfully” and “I feel comfortable approaching our school's police officer with a problem.” Items were measured on a 4‐point Likert‐scale (*strongly agree* to *strongly disagree*), and were reverse coded as needed so that a higher scale score was associated with more perceived caring (5‐item α = .93).

#### *Control variable*

*Family racial socialization* was measured using the Teenager Experience of Racial Socialization scale.^51^ On a 4‐point Likert‐scale, students were asked the following question, “do your parents or any of your caregivers say to you any of the following statements now, or when you were younger?” and then instructed to check the box indicating how often (never, sometimes, often, very often) they remembered hearing the given messages. Statements included: “be proud of who you are,” “education is the only way to survive racism,” and “racism and discrimination are hard to face” (6‐item α = .80).

### Procedure

As noted above, we leveraged student‐reported baseline data from a school‐based violence prevention intervention in 10 urban high schools in a larger mid‐Atlantic city. Only baseline data were utilized to avoid confounding of intervention effects in this secondary data analysis. A teacher screening procedure was used to determine students' eligibility for participation, whereby teachers universally screened all ninth‐grade students for indicators of aggression using a 6‐item measure that focused on reactive and proactive aggressive behaviors.^52^ Approximately 5800 ninth‐graders were screened. Thereafter, ninth‐grade students were eligible for enrollment based on their ranking in the top 35% of ninth‐grade students demonstrating the most acute aggressive behaviors (based on a cutoff score of 9).^53^ Project staff contacted parents/guardians of students to obtain written informed consent and youth assent, resulting in 514 students enrolled in the study.

### Data Analysis

**Figure 1:**
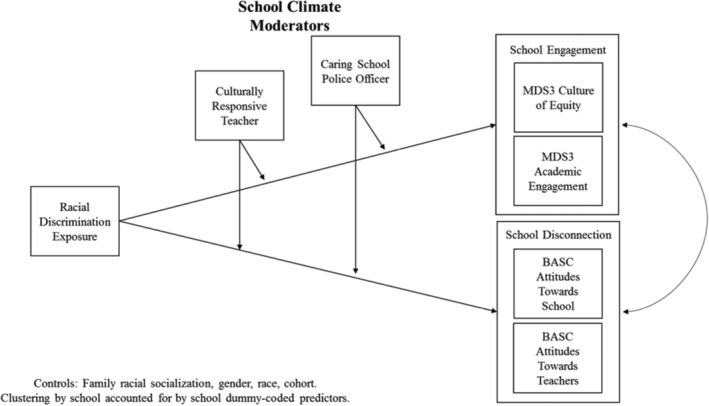
Path Diagram for Moderation of Culturally Responsive Teacher Practices and Caring School Police Officer

We fit a path model to the data using robust, full‐information maximum likelihood estimation in M*plus* 8 software,^54^ to account for any missing data as well as any multivariate non‐normality. In a single path model, we regressed the 4 outcomes, school culture of equity, academic engagement, school disconnection, and teacher disconnection on covariates and interaction terms of interest (Figure 1). We fit one model to the data that was supported by theorized relationships in the literature. Although not depicted in the simplified path diagram, all outcome residuals were free to correlate as were all predictor variables.

Frequency of racial discrimination was included as the focal predictor. Teacher CRPs and caring school police variables were the hypothesized moderators and included as predictors in the model. Interaction terms were created by multiplying the frequency of discrimination variable by each of the hypothesized moderator variables (ie, teacher CRPs and caring school police officer variables) and included in the model to elucidate whether the relationships between racial discrimination and the outcomes were significantly moderated. In addition to including these key predictors, we also controlled for family racial socialization, sex, race, and study cohort. All continuous predictors were centered before creating interaction terms and to aid in interpretability of the model intercepts. The students were nested within 10 schools, which is too few to utilize a multilevel modeling approach or cluster‐robust standard errors, so we utilized a fixed‐effects approach to model the nesting within schools as recommended in the methodological literature.^55^, ^56^

## RESULTS

### School Engagement

Table 2 presents the results of the path model. The model accounted for 21.1% of the variance in academic engagement and 39.6% of the variance in the culture of equity subscale. Male students demonstrated lower levels of academic engagement than female students (β = −0.102, p = .042), but there was not a significant impact of sex on the Culture of Equity subscale. As hypothesized, greater frequency of racial discrimination was associated with lower academic engagement (β = −0.127, p = .024) and lower culture of equity (β = −0.194, p = .001). Alternatively, higher family racial socialization was associated with greater academic engagement (β = 0.278, p < .001) and culture of equity (β = 0.195, p < .001). Teacher CRPs were also associated with increased academic engagement (β = 0.255, p < .001) and an increased culture of equity (β = 0.474, p < .001). The effects of a caring school police officer and the interaction terms were non‐significant.

**Table 2:** Path Model Results

	School Engagement						School Disconnection					
	Academic Engagement			Culture of Equity			Teacher Attitudes			School Attitudes		
Predictor	β	SE	p	β	SE	p	β	SE	p	β	SE	p
Sex (male = 1)	−0.102	0.050	.042	0.041	0.048	.392	−0.010	0.050	.836	0.001	0.048	.985
Race (Black = 1)	−0.050	0.055	.367	−0.029	0.041	.484	0.011	0.049	.818	0.081	0.045	.069
Family racial socialization	0.278	0.057	<.001	0.195	0.043	<.001	−0.136	0.053	.011	−0.121	0.059	.040
Racial Discrimination	−0.127	0.056	.024	−0.194	0.056	.001	0.185	0.053	.001	0.081	0.052	.123
School police caring	0.061	0.058	.289	0.093	0.050	.060	−0.002	0.070	.976	−0.062	0.072	.384
Teacher CRPs	0.255	0.053	<.001	0.474	0.052	<.001	−0.310	0.059	<.001	−0.292	0.058	<.001
Discrimination × Police Caring	−0.024	0.051	.637	−0.001	0.046	.990	−0.046	0.060	.443	−0.169	0.064	.008
Discrimination × Teacher CRPs	−0.048	0.054	.378	−0.077	0.057	.178	0.023	0.061	.709	0.077	0.057	.176
R^2^	0.211			0.396			0.207			0.217	

### School Disconnection

The model accounted for 20.7% of the variance in attitudes toward teachers and 21.7% of the variance in attitudes toward school. As hypothesized, greater frequency of racial discrimination was associated with worse attitudes toward teachers (β = 0.185, p < .001), but was not significantly related to attitudes toward school. Increased family racial socialization was associated with more positive attitudes toward teachers (β = −0.136, p = .011) and school (β = −0.121, p = .040). Teacher CRPs were also associated with better attitudes toward teachers (β = −0.310, p < .001) and school (β = −0.292, p < .001).

**Figure 2:**
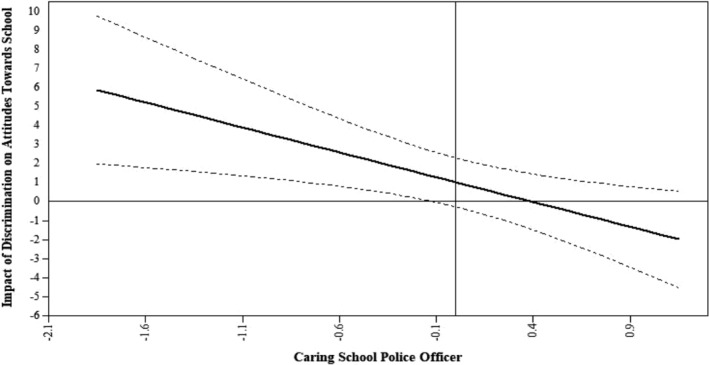
Johnson‐Neyman Plot of the Relationship between Frequency of Discrimination and Attitudes toward School across Levels of Caring School Police Officer

The effects of a caring school police officer and the interaction terms were non‐significant with one exception. The interaction between discrimination and caring school police was a significant predictor of attitudes toward school (β = −0.169, p = .008). Figure 2 presents the Johnson‐Neyman plot^57^ for the interaction between frequency of racial discrimination and the caring school police officer assessment. This plot presents the conditional effect of our focal predictor, frequency of discrimination, on the outcome, attitudes toward school, across all possible values of the moderator, caring school police. Our results suggest that when police officer caring was low, more experiences of racial discrimination were associated with poorer attitudes toward school. However, when police officer caring was in the average to high range, there was no significant association of discrimination with attitudes toward school.

## DISCUSSION

Several key findings emerged regarding the association between perceived discrimination, school engagement, and disconnection, and the moderating role of teacher cultural responsiveness and school police caring. Overall, the models explained about 20% of the variance in 3 of the outcomes (ie, attitudes toward school and teachers and academic engagement); for school culture of equity, the model explained almost 40% of the variance. As hypothesized, we found that perceived frequency of discrimination, including personal experiences, witnessing, hearing about, and observing societal forms of discrimination, was associated with lower levels of school engagement and more negative attitudes toward teachers. Since the measure assessing the frequency of discrimination was not setting‐specific, this finding suggests that regardless of whether the school was a source of perceived discrimination, such experiences were nonetheless significantly associated with students' perceptions of school engagement and attitudes toward their teachers. The finding is also consistent with prior research on societal discrimination as it relates to school contexts,^26^ and further extends it by establishing links between general exposure to racial discrimination that occurs across settings (personal, family and neighborhood, and societal, not specific to the school), with clinical indicators of school disconnection.

Our analyses also suggested some protective relationships for these youth, which may be particularly important to consider given their elevated risk for engaging in aggressive and disruptive behaviors. Although not a central focus of the current study, family racial socialization practices were significantly associated with all 4 school engagement and school disconnection outcome measures, confirming prior research that family communication about racial pride and racialized life experiences has an important function in students' developmental outcomes and wellbeing.^3^ Moreover, controlling for racial socialization and other covariates, we found protective and promotive school relationships in the school setting. We found that across all 4 outcome measures, teacher cultural responsiveness was associated with more school engagement and less school disconnection, suggesting a potential promotive effect consistent with research on cultural responsiveness^32^; however, we did not find evidence of a *protective* effect of teacher cultural responsiveness (ie, no significant attenuation of the association between perceived discrimination on any of the outcomes was found).

On the other hand, no promotive associations of school police caring were found on any of the outcomes, aligned with prior research showing the lack of an association between school police and student behaviors,^40^ but inconsistent with prior research showing school police presence was linked to lower levels of school connectedness.^41^ However, school police caring did attenuate the association between perceived discrimination and negative school attitudes, suggesting that relationships with school police, when perceived as positive by students, may play a role in mitigating the negative effects of perceived discrimination on school disconnection. This finding is timely to consider in light of national protests following the murder of George Floyd in summer 2020, which have prompted shifts in the national dialogue on the topic of investment in policing, including policing in schools.^58^ Some school districts, under increased pressure to defund the police, have terminated school resource officer contracts and divested in policing in schools.^59^ These decisions are well‐justified, as research suggests that the presence of police in schools increases likelihood of student arrest for minor offenses (ie, disorderly conduct) and exclusionary discipline,^60^, ^69^ which can set youth on harmful trajectories consistent with concerns about the criminalization of student conduct in the classroom and the school to prison pipeline.^61^

It is also important to consider that some statewide studies of high school student perceptions of school police in Maryland and Virginia suggest that students find the presence of school police officers reassuring and associate police presence with a sense of safety and support.^42^, ^62^ However, in one study, this pattern differed by race, whereby the frequency of students disagreeing that school police made them feel safer was higher among black students than white students.^62^ The other study also showed a reduced association of officer presence with black students' perceptions of school safety relative to white students, though the latter finding was marginal.^42^ Given these considerations, the current study's findings require careful interpretation in context.

Specifically, although demographic data on officers specifically stationed at the schools in this study are unavailable, available demographic data on a convenience sample of officers in the district suggest the officer sample was likely to be about 75% male and over 90% black. In these data, a little under half of the officers had combined annual household incomes under $80,000 and about 80% of officers grew up and/or lived as adults in the same urban community where the students attended school. Officers were also experienced in school settings; 75% of officers reported being school police officers for over 9 years.^63^ Anecdotally, a number of officers in the study functioned in an informal counselor capacity in addition to their law enforcement duties, providing mentoring programs, food and clothes banks for students before school, and other supports for students, usually on their own time. Thus, officers were not only racially representative of students in the school, but also had similar experiences in terms of lived experience in the same city and extensive time in school settings; they may also have functioned in a more supportive role than is typical. It may be that the findings in this study would differ if there were greater sociocultural and ecological distance between officers and students (ie, cross‐racial mismatch and mistrust, officers living outside of the city). More substantive and diverse data on school police officers is needed to generalize these findings to other settings.

### Strengths and Limitations

There are many strengths of this study, including its representation of the perspectives of student stakeholders, and particularly those of students of color screened with elevated risk of aggression and clinically derived measures of school disconnection. Students with elevated risk of aggression are more likely to come into contact with school police than the general student population, and thus are a population that merits research attention to inform prevention efforts. We also take a strengths‐based approach in our focus on assets for urban, black youth resilience as it relates to school engagement. A novel element of the study was the focus on school police, as these relationships have rarely been accounted for in studies of school engagement, particularly among high‐risk students. This study contributes to a small but growing literature base on engagement related outcomes of CRPs and interactions with school police officers.

Despite these strengths, there are some limitations to consider. For example, students' baseline risk and eligibility for inclusion in the project was assessed by classroom teachers, and thus may have been subject to bias or not have adequately detected all students in need of support. Relatedly, the screening data contained measures of the types of aggression (proactive, reactive); however, our IRB approval did not include use the screening data in these analyses. This is because the screening measure was universally administered and as such different consent procedures applied relative to our data collected in the context of the trial. As such, we were not able to assess whether there were differences across proactive versus reactive aggression profiles. In addition, we did not account for measurement error in our modeling approach. Modeling each of the predictors and outcomes as a latent variable would have added many more parameters and complexity to the model than were plausible given the scope of our study and the capacity of our sample size. Countering this concern, we utilized measures that have been well‐validated and also demonstrated adequate reliability in our sample. Although we do not believe that these associations were severely attenuated due to unreliability, future research with larger and more diverse samples should consider a modeling approach that takes measurement error into account, such as using a latent variable modeling approach. Another limitation is that our measure of discrimination frequency was not specific to the source of students' reported experiences of discrimination; it does not indicate school specifically, but it also did not preclude school experiences. Greater precision in the measure on the source of racial discrimination might have furthered our ability to make inferences about the role of school and non‐school‐related discrimination on school outcomes. The findings, based on this sample of predominantly black students with elevated risk of aggression in urban public schools, may not generalize to other student groups. Furthermore, our data are cross‐sectional, so causal inferences cannot be made, and rely heavily on students' self‐reports. For example, it may be that students' who feel more engaged at school perceive higher levels of teacher CRPs, rather than the other way around, as hypothesized in this study.

## IMPLICATIONS FOR SCHOOL HEALTH

Taken together, these findings contribute to the literature on how teachers and school police officers, through their use of culturally responsive and caring practices, may promote school engagement and minimize disconnection. In addition, family racial socialization practices were significantly, favorably associated with all 4 school engagement and school disconnection outcomes. Based on the findings in this study, we elaborate on corresponding implications for schools as a result of this research.

### Implication 1: Training for School Police

We found that, given experiences of racial discrimination, positive interactions with caring school police officers were a protective factor in this sample of urban, public school students of color with elevated rates of externalizing symptoms. These results suggest that training for school police that increases their levels of caring may mitigate the effects of discrimination on school disconnection. In addition, students may perceive school police officers to be more caring when they are engaging in the informal counselor role identified by the National Association of School Resource Officers. As such, relevant trainings might include a focus on practicing empathy and perspective‐taking, building awareness of positive youth development, and increasing sensitivity to youth mental and behavioral health needs.

#### *Practice example*

For example, Youth Mental Health First Aid training teaches professionals to recognize symptoms of mental and behavioral health problems, respond supportively to students in distress, and identify appropriate resources for them.^64^ This training promotes participants mental health literacy and provides an appropriate levels of support, especially for practitioners who have little other training in youth mental health needs.^65^, ^66^ Mental Health First Aid training may be able to increase school police officers' abilities to function in an informal counselor role, which may positively impact students' perceptions of school police officers as caring.

### Implication 2: Increase Teacher Capacity to Implement CRPs

Similarly, our results suggest that training and coaching for teachers that increase their usage of CRPs in the classroom relates to school engagement and may minimize school disconnection. School and district‐level leaders may consider implementing training and professional development to increase teacher capacity to engage in CRPs in the classroom. Empirical research on the effectiveness of interventions to promote teacher cultural responsiveness and reduce disparate treatment of students is emerging.^34^

#### *Practice example*

For example, the Double Check intervention, which includes group professional development and coaching for teachers to increase their use of CRPs, has been found to improve independently observed effective classroom behavior management and teacher self‐reported cultural responsiveness.^36^ Coordination of these training and programmatic efforts in a student‐centered framework, such as the WSCC^8^ could help to promote equitable school staff practices and positive student engagement for at‐risk students.

### Implication 3: Family Engagement

Finally, family racial socialization practices were significantly associated with school engagement and disconnection. While this is inherently a family‐driven practice, schools may offer resources (facilities, professional staff, existing structures) to facilitate intervention to help promote this protective family practice.

#### *Practice example*

Students demonstrating elevated levels of aggressive behavior may be invited, along with their caregiver(s), to participate in a tier 2 or tier 3 preventive intervention as part of a schoolwide universal prevention program. Such interventions typically do not include an element on racial socialization, unfortunately, despite black caregivers having identified racial socialization as an important parenting skill. Incorporation of these practices into existing evidence‐based parenting programs may increase the cultural responsiveness of these interventions for black families.^67^ Initial evaluations of a dyadic parent‐child racial socialization intervention, EMBRACE (Engaging, Managing, and Bonding through Race, have shown promise in increasing family racial socialization practices and decreasing racial stress.^68^

### Human Subjects Approval Statement

All data collection procedures were approved by the Institutional Review Boards at the school district, participating universities, and partner organization.

### Conflict of Interest

All authors of this article declare they have no conflicts of interest.
